# IL-15 Fosters Age-Driven Regulatory T Cell Accrual in the Face of Declining IL-2 Levels

**DOI:** 10.3389/fimmu.2013.00161

**Published:** 2013-06-24

**Authors:** Jana Raynor, Allyson Sholl, David R. Plas, Philippe Bouillet, Claire A. Chougnet, David A. Hildeman

**Affiliations:** ^1^Division of Cellular and Molecular Immunology, Department of Pediatrics, Cincinnati Children’s Hospital Medical Center, University of Cincinnati, Cincinnati, OH, USA; ^2^Department of Cancer and Cell Biology, University of Cincinnati, Cincinnati, OH, USA; ^3^Molecular Genetics of Cancer Division, Walter and Eliza Hall Institute of Medical Research, Melbourne, VIC, Australia; ^4^Department of Medical Biology, University of Melbourne, Melbourne, VIC, Australia

**Keywords:** CD25, Bim, IL-2, IL-15, aging, T_reg_

## Abstract

We and others have shown that regulatory T cells (T_reg_) accumulate dramatically with age in both humans and mice. Such T_reg_ accrual contributes to age-related immunosenescence as they reduce the response to tumors and parasite infection. While we reported earlier that aged T_reg_ have decreased expression of the pro-apoptotic molecule Bim and germline deletion of Bim promoted earlier accumulation of T_reg_, it remains unclear whether the effects of Bim are: (i) T_reg_ intrinsic and (ii) dominant to other BH3-only pro-apoptotic molecules. Further, the mechanism(s) controlling Bim expression in aged Treg remain unclear. Here we show that T_reg_-specific loss of Bim is sufficient to drive T_reg_ accrual with age and that additional loss of the downstream apoptotic effectors Bax and Bak did not exacerbate T_reg_ accumulation. Further, our results demonstrate that a subpopulation of T_reg_ expands with age and is characterized by lower expression of CD25 (IL-2Rα) and Bim. Mechanistically, we found that IL-2 levels decline with age and likely explain the emergence of CD25^lo^Bim^lo^ T_reg_ because T_reg_ in IL-2^−/−^ mice are almost entirely comprised of CD25^lo^Bim^lo^ cells, and IL-2 neutralization increases CD25^lo^Bim^lo^ T_reg_ in both young and middle-aged mice. Interestingly, the T_reg_ population in aged mice had increased expression of CD122 (IL-2/IL-15Rβ) and neutralization or genetic loss of IL-15 led to less T_reg_ accrual with age. Further, the decreased T_reg_ accrual in middle-aged IL-15^−/−^ mice was restored by the additional loss of Bim (IL-15^−/−^Bim^−/−^). Together, our data show that aging favors the accrual of CD25^lo^ T_reg_ whose homeostasis is supported by IL-15 as IL-2 levels become limiting. These data have implications for manipulating T_reg_ to improve immune responses in the elderly.

## Introduction

Aging is associated with declining immune function, which contributes to increased infectious diseases, increased cancer, and decreased vaccine efficacy in the elderly. The aging immune system is characterized by a progressive deterioration in the adaptive immune system, particularly affecting T cells (Linton and Dorshkind, 2004; Maue et al., 2009). Factors contributing to T cell dysfunction with age include: (i) thymic involution and decreased naïve T cell production (Sempowski et al., 2002; Hale et al., 2006); (ii) impaired TCR signaling and immune synapse formation (Miller et al., 1997; Miller, 2000; Tamir et al., 2000; Garcia and Miller, 2002); (iii) impaired T cell proliferation (Murasko et al., 1987; Haynes et al., 2005); (iv) CD8^+^ T cell clonal expansion driven by chronic infections (Khan et al., 2002; Ouyang et al., 2003; Clambey et al., 2005). While many of these effects are T cell intrinsic, recent work has found that regulatory T cell (T_reg_) frequencies increase dramatically with age in both mice and humans and may contribute substantially to impaired T cell responses in aged hosts (Valmori et al., 2005; Nishioka et al., 2006; Sharma et al., 2006; Lages et al., 2008; Agius et al., 2009).

Regulatory T cells, a specialized subset of CD4^+^FoxP3^+^ T cells, are known to control the intensity of immune responses through modulating the activation and function of both effector T cells and antigen-presenting cells (Miyara and Sakaguchi, 2007; Onishi et al., 2008). The functionality of aged T_reg_ have not been well studied, however we have shown that aged FoxP3^+^ T_reg_ have an equal or increased *in vitro* suppressive capacity compared to young T_reg_ (Lages et al., 2008). *In vivo*, depletion of CD25^+^ T_reg_ allowed for a more robust CD4^+^ T cell response against *Leishmania major* in aged mice, suggesting increased T_reg_ in the aged can dampen effector T cell activation (Lages et al., 2008). Additionally, T_reg_ accrual with age has been shown to inhibit anti-tumor responses (Sharma et al., 2006). Thus, aged T_reg_ appear to be functional *in vivo* and T_reg_ accrual may contribute significantly to immunosenescence in aging.

Many studies have looked at the factors involved in T_reg_ homeostasis in young mice, particularly the γc cytokines IL-2, IL-7, and IL-15. The receptor for IL-2 is comprised of CD25 (IL-2Rα), CD122 (IL-2/15Rβ), and CD132 (IL-2/15/7Rγ). IL-2 shares the IL-2/15Rβ receptor with IL-15, and the γc receptor (CD132) with both IL-15 and IL-7. IL-2 is the dominant cytokine required for T_reg_ survival and homeostasis, as the loss of IL-15 or IL-7 signaling does not substantially affect the frequency of CD4^+^ cells that are T_reg_ when IL-2 is present (Burchill et al., 2007; Bayer et al., 2008; Vang et al., 2008). However, CD132 and to a lesser extent CD122-deficient mice have a more profound loss of T_reg_ compared to IL-2 or CD25 deficient mice, suggesting that IL-15 and/or other γc cytokines also contribute to T_reg_ homeostasis (Fontenot et al., 2005a). All of these studies examining the requirements for cytokine signaling in T_reg_ development and survival have been done in young mice, and the role for the γc cytokines in aged T_reg_ homeostasis is unclear.

Our previous study showed that Bim plays a major role in T_reg_ homeostasis and that Bim levels decline significantly in aged T_reg_ (Chougnet et al., 2011). Further germline deletion of Bim led to significantly faster accrual of T_reg_ (Chougnet et al., 2011). Here, we found that T_reg_-specific loss of Bim was sufficient to drive T_reg_ accrual and that Bim was the dominant pro-apoptotic molecule driving T_reg_ accrual. Further, decreased Bim levels in aged T_reg_ is reflected by decreased Bim mRNA and increased Bim turnover. Additionally, declining IL-2 levels with age resulted in reduced levels of CD25 and increased levels of CD122 which foster T_reg_ dependence upon IL-15, which, in turn, functions to restrain the remaining Bim in aged T_reg_.

## Materials and Methods

### Mice

C57BL/6 mice were purchased from either Taconic Farms (Germantown, NY, USA) or the National Institutes of Aging colony located at Charles River Laboratories (Wilmington, MA, USA). B6.129P2-*Il2^*tm1Hor*^*/J (IL-2^−/−^) mice and their C57BL/6 controls were purchased from The Jackson Laboratory (Bar Harbor, ME, USA). B6.SJL-*Ptprc^*a*^ Pepc^*b*^*/BoyJ mice on the C57BL/6 background were purchased from The Jackson Laboratory and aged in house. Bim^−/−^ mice have been backcrossed to C57BL/6 mice for at least 20 generations. Bim^f/f^ mice were generated at the Walter and Eliza Hall Institute as part of a collaborative effort with Dr. P. Bouillet. Briefly, a targeting vector was created by flanking coding exons 2, 3, and 4 of *Bim* with loxP sites. The vector was electroporated into C57BL/6 embryonic stem (ES) cells and homologously recombined ES cells were selected with hygromycin. The hygromycin cassette was removed by crossing the Bim^f/f^ mice with B6.Cg-Tg(ACTFLPe)9205Dym/J (Jackson Labs) to generate a Bim floxed allele that could be crossed to tissue – specific cre transgenic mice to achieve tissue-specific deletion of Bim. Offspring from this cross were screened for removal of the Hygromycin cassette and maintenance of the conditional Bim allele. Mice were then bred to Cre-expressing mice and offspring screened for lack of the ACTFLPe allele. Lck-Cre Bax^f/f^Bak^−/−^ mice were a gift from Dr. S. Korsmeyer and were previously described (Takeuchi et al., 2005). IL-15-deficient mice on the C57BL/6 background were purchased from Taconic Farms, mated with the Bim^−/−^ mice to generate IL-15^−/−^ Bim^−/−^ mice, and aged in house. FoxP3-IRES-DTR-GFP knock-in C57BL/6 mice (Kim et al., 2007) and FoxP3-Cre mice (Rubtsov et al., 2008) were a gift from Dr. A. Rudensky. FoxP3-IRES-DTR-GFP mice were aged in house. FoxP3-Cre mice were mated with Bim^f/f^ mice and aged in house. Mice were housed under specific pathogen-free conditions. All animal protocols were reviewed and approved by our Institutional Animal Care and Use Committee.

### Flow cytometry

Spleens, lymph nodes (inguinal, axillary, and brachial), and thymi were harvested and crushed through 100 μm filters (BD Falcon) to generate single-cell suspensions. About 1 × 10^6^ cells were surface stained with Abs against CD4 (BD Biosciences, San Diego, CA, USA), CD25 (eBioscience, San Diego, CA, USA), CD44 (eBioscience), CD122 (Biolegend, San Diego, CA, USA), CD45.2 (eBioscience) and intracellularly for FoxP3 (eBioscience), Bim (Cell Signaling Technology, Beverly, MA, USA), and Ki67 (eBioscience). All intracellular stains were performed using the eBioscience FoxP3 staining protocol. For detection of Bim, secondary anti-rabbit IgG Abs were used (Jackson ImmunoResearch Laboratories, West Grove, PA, USA; Invitrogen, Carlsbad, CA, USA; or Cell Signaling Technology). Data were acquired on an LSRII flow cytometer (BD Biosciences), analyzed using FACSDiva software (BD Biosciences), and histogram overlays were prepared using FlowJo software (Tree Star).

### Adoptive transfers

Spleen cells from young (3–4 months) and old (19–23 months) FoxP3-IRES-DTR-GFP mice were enriched for CD4^+^ cells using the negative selection MACS CD4^+^ T cell Isolation Kit II (Miltenyi Biotec, Auburn, CA, USA) and stained with CD4 antibody. CD4^+^FoxP3^GFP+^ cells were then sorted by FACSAria (BD Biosciences), and>85% purity was obtained. About 5 × 10^5^ cells were injected i.v. into young (2 months) or aged (15 months) C57BL/6 congenic CD45.1 recipient mice. The recipient mice were sacrificed either 1.5 or 10 days post-transfer, spleens were harvested, and single-cell suspensions were stained for CD4, CD45.2, CD25, and intracellularly for FoxP3. Cells were fixed with 4% methanol-free formaldehyde (Polysciences, Warrington, PA, USA), instead of the eBioscience FoxP3 fix-perm buffer, to better retain GFP within the cells.

### *In vivo* cytokine capture assay

IL-2 *in vivo* cytokine capture assay (IVCCA) was performed as described (Finkelman and Morris, 1999; Finkelman et al., 2003). Briefly, young (2–5 months), middle-aged (11–12 months), and old (>15 months) C57BL/6 mice were injected i.v. with 10 μg of biotinylated anti-IL-2 capture antibody (JES6-5H4-BD Biosciences), or with PBS as a control, and mice were bled 24 h later and serum was collected. Ninety-six-well Costar plates were coated overnight with anti-IL-2 JES61A12 (eBioscience) and then a luminescent ELISA was performed. For the cytokine:anti-cytokine mAb standard, 100 ng of recombinant mouse IL-2 (R&D) was incubated with 10 μg of the anti-IL-2 capture antibody (JES6-5H4) for 10 min, and 100 ng/ml aliquots were stored in −80° C freezer. The IL-2 concentration obtained from our PBS control was subtracted from our young and old samples to remove the ELISA background.

### *In vivo* cytokine neutralization

Anti-IL-2 Ab (clones S4B6 and Jes61A12) and rat IgG2A isotype control (2A3) were purchased from BioXcell (West Lebanon, NH, USA). Anti-IL-15 (M96) was a kind gift from Amgen (Seattle, WA, USA). For IL-2 neutralization: 3- and 12-month-old mice were injected i.p. with 170 μg of S4B6 and Jes61A12, or with isotype control (2A3), on days 0, 1, 2, 4, 6, and sacrificed on day 7 and spleens were harvested. For IL-15 neutralization: 3- and 12-month-old mice were injected i.p. with 25 μg of M96 on days 0, 2, 4, 6, and sacrificed on day 7. IL-15 neutralization was confirmed by assessing natural killer cells, which showed>60% deletion in the spleen (data not shown).

### Cycloheximide assay

Splenocytes from either young (3 months) or old (22 months) C57BL/6 mice were cultured with or without cycloheximide (20 μM; Sigma-Aldrich, St. Louis, MO, USA) for 8 h at 37° C. Cells were stained for CD4, FoxP3, and Bim and analyzed by flow cytometry. Percent decrease in Bim expression was determined by comparing Bim expression between cells cultured with our without cycloheximide.

### Quantitative real-time PCR

Spleen cells from young (3 months) and old (22 months) FoxP3-IRES-DTR-GFP mice were enriched for CD4^+^ cells using the negative selection MACS CD4^+^ T cell Isolation Kit II (Miltenyi Biotec) and stained with CD4 antibody. CD4^+^FoxP3^GFP+^ and CD4^+^FoxP3^GFP−^ cells were then sorted by FACSAria (BD Biosciences) and>90% purity was obtained for both populations. RNA was isolated from cells using the RNeasy Mini Kit (Qiagen) and converted into cDNA using Superscript II Reverse Transcriptase (Invitrogen, CA, USA). The primers used for Bim were: 5′-ACAAACCCCAAGTCCTCCTT-3′ and 5′-GTTTCGTTGAACTCGTCTCC-3′; and for the internal control L19: 5′-CCTGAAGGTCAAAGGGAATGTG-3′ and 5′-GCTTTCGTGCTTCCTTGGTCT-3′. Quantitative real-time PCR was performed with Roche LightCycler 480 SYBRGreen 1 Master Mix using the Roche LightCycler 480 II instrument (Roche Diagnostics).

## Results

### Bim progressively decreases in T_reg_ with age

**Figure 1:**
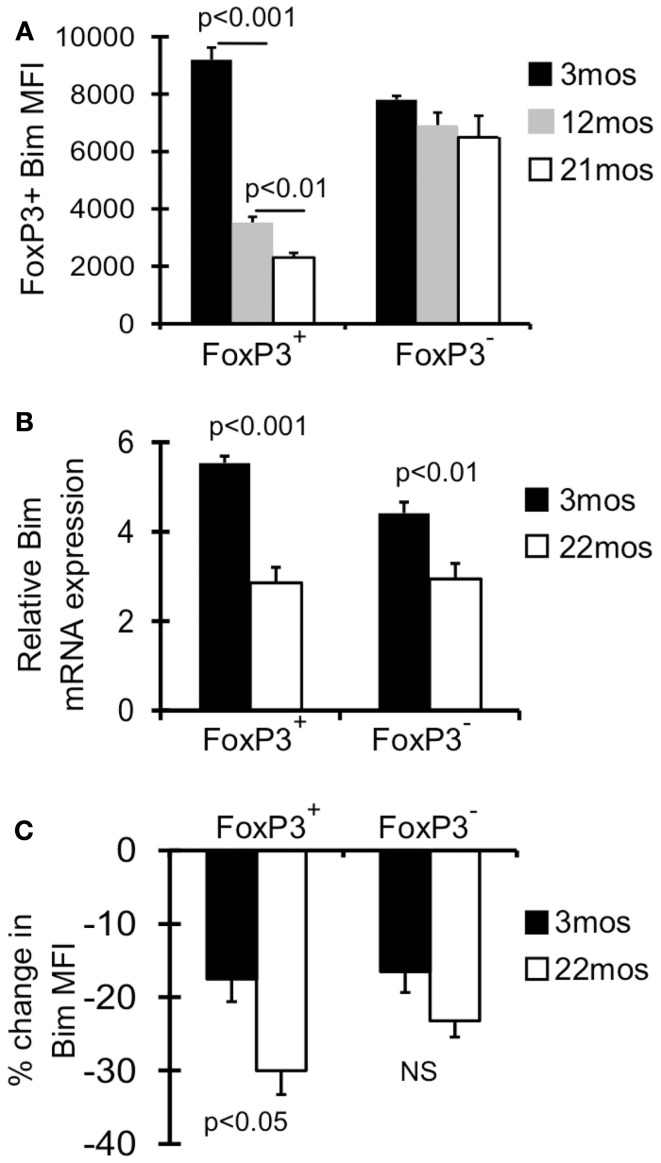
**Bim levels in T_reg_ are progressively decreased with age**. **(A)** Splenocytes from 3 month (*n* = 4), 12 month (*n* = 4), and 21-month-old (*n* = 5) mice were stained with Abs against CD4, FoxP3, and Bim and analyzed by flow cytometry. Results show the average mean fluorescent intensity (MFI) of Bim in CD4^+^FoxP3^+^ T cells (±SE). The *p* values represent the difference between 3 and 12 months; 12 and 21 months (Student’s *t* test). **(B)** FoxP3^GFP^ reporter mice were used to sort CD4^+^FoxP3^GFP+^ T_reg_, CD4^+^FoxP3^GFP−^ non-T_reg_ from young (3 months; *n* = 4) and old (22 months; *n* = 4) mice. Results show the relative expression of Bim mRNA in 3 and 22 months mice (±SE). The *p* values represent the difference between 3 and 22 months (Student’s *t* test). **(C)** Splenocytes from 3 months (*n* = 4) and 22 months (*n* = 3) mice were cultured with or without cycloheximide for 8 h at 37° C, and then stained for CD4, FoxP3, and Bim and analyzed by flow cytometry. Results show the percent decrease in Bim expression within CD4^+^FoxP3^+^ and CD4^+^FoxP3− cells cultured with cycloheximide (±SE). Results are representative of at least two independent experiments.

We have previously shown that T_reg_ in aged mice have decreased expression of Bim, and Bim^−/−^ mice accrued T_reg_ more rapidly, together suggesting loss of Bim expression drives T_reg_ accrual with age (Chougnet et al., 2011). Here we investigated this in more detail and found that the decrease in Bim expression with age is a progressive process (Figure 1A). The major loss in Bim expression is observed by 12 months (threefold; fourfold by 21 months), and most significantly within the CD4^+^FoxP3^+^ population, not in the CD4^+^FoxP3− population (Figure 1A) (Chougnet et al., 2011).

As Bim is subject to transcriptional, translational, and post-translational control, we next determined the relative contribution of these mechanisms. First, CD4^+^FoxP3^GFP+^ T_reg_ and CD4^+^FoxP3^GFP−^ non-T_reg_ were sorted from young (3 months) and old (22 months) FoxP3^GFP^ reporter mice (Kim et al., 2007). T_reg_ cells from old mice showed significantly decreased Bim mRNA (∼2-fold), which was observed to a lesser extent in non-T_reg_ (Figure 1B). Next, to examine Bim protein turnover, splenocytes from young and old mice were cultured with cycloheximide, an antibiotic that inhibits translation. Interestingly, old FoxP3^+^ T_reg_ cells had significantly greater Bim turnover compared to young T_reg_ (30 vs. 18%, respectively; Figure 1C). These data indicate that both decreased Bim transcription and increased Bim turnover likely contribute to the decreased Bim protein levels observed with age.

### T_reg_ intrinsic loss of Bim expression promotes accrual with age

**Figure 2:**
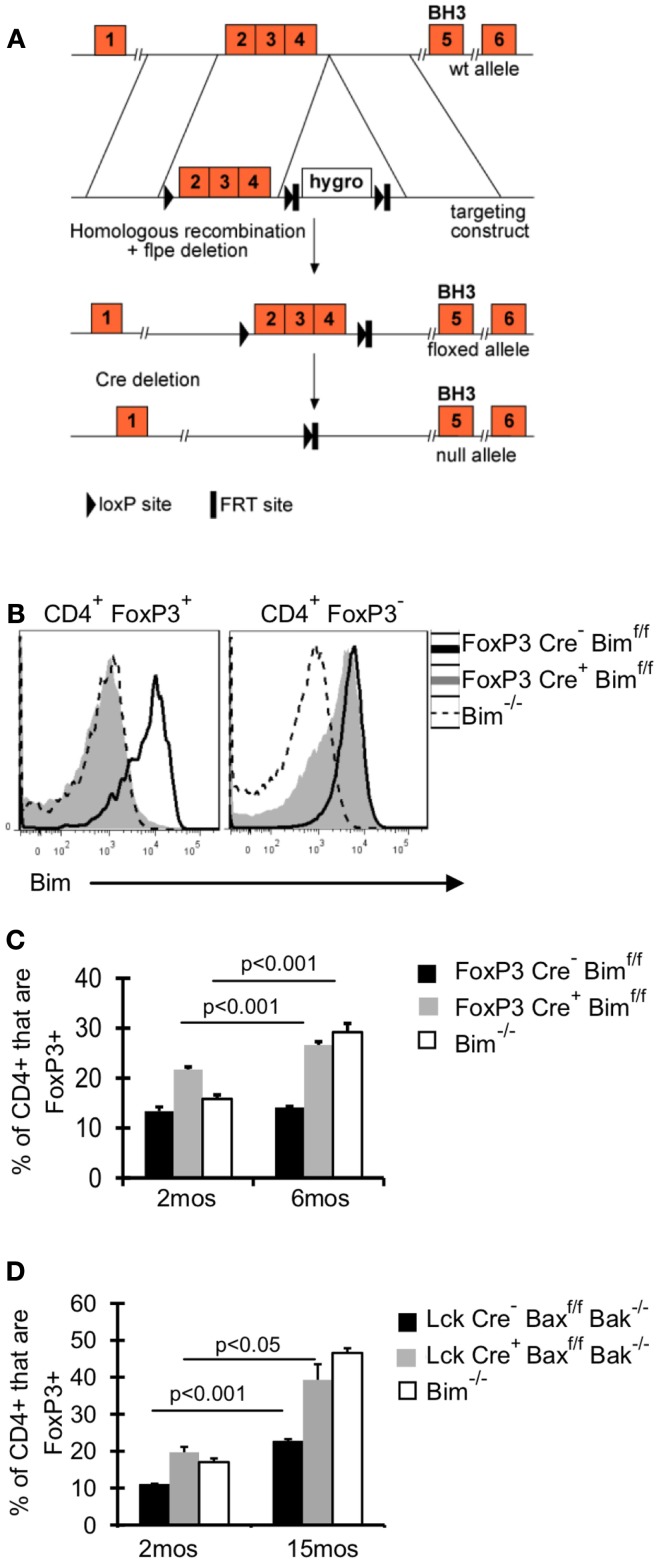
**Bim is the dominant BH3-only molecule in T_reg_ and its effects on T_reg_ homeostasis are T_reg_ intrinsic**. **(A)** A conditional *Bim* allele was generated by targeting loxP sites upstream of exon 2 and downstream of exon 4. **(B,C)** Cells from the lymph nodes of 2- and 6-month-old FoxP3-Cre^−^ Bim^f/f^, FoxP3-Cre^+^Bim^f/f^, and Bim^−/−^ mice were stained for CD4, FoxP3, and Bim and analyzed by flow cytometry. **(B)** Histograms show the expression of Bim in CD4^+^FoxP3^+^ and CD4^+^FoxP3^−^ T cells from 2-month-old mice. **(C)** Results show the frequency of CD4^+^ cells that are FoxP3^+^ in 2 month (*n* = 5/group) and 6-month-old mice (*n* = 3–4/group; ±SE). The *p* values represent the difference between 2 and 6 months (Student’s *t* test). Results are representative of two independent experiments. **(D)** Splenocytes from Lck-Cre^−^Bax^f/f^Bak^−/−^(*n* = 3–5/group), Lck-Cre^+^Bax^f/f^Bak^−/−^(*n* = 3–5/group), and Bim^−/−^ (*n* = 3–4/group), mice were stained for CD4, FoxP3, and Bim and analyzed by flow. The Bim^−/−^ mice were analyzed in an independent experiment. Results show the frequency of CD4^+^ cells that are FoxP3^+^ in 2- and 15-month-old mice (±SE). The *p* values represent the difference between 2 and 15 months (Student’s *t* test).

It remains unclear whether the effects of Bim on T_reg_ accrual are T_reg_ intrinsic. To address this question, we crossed FoxP3-Cre mice (Rubtsov et al., 2008) with mice that have loxP flanked Bim on both alleles (Bim^f/f^) (Figure 2A), and aged these FoxP3-Cre Bim^f/f^ mice to 6 months. Bim staining in FoxP3-Cre^+^Bim^f/f^ mice was comparable to Bim^−/−^ mice, confirming efficient deletion of Bim (Figure 2B). There was a small fraction of CD4^+^FoxP3^–^ cells with somewhat reduced expression of Bim, suggesting that some cells may have deleted one or both alleles of Bim (Figure 2B). However, we did not observe loss of Bim in non-T cell populations (i.e., CD11c^+^ or B220^+^ cells, data not shown). The frequency of T_reg_ in FoxP3-Cre^+^Bim^f/f^ mice was increased at 2 months of age, compared to either FoxP3-Cre^–^ Bim^f/f^ or Bim^−/−^ mice (Figure 2C). This increase in T_reg_ at 2 months of age may be due to increased thymic output in FoxP3-Cre^+^Bim^f/f^ mice, as we see increased frequencies and numbers of FoxP3^+^ cells in the thymi of FoxP3-Cre^+^Bim^f/f^ mice compared to FoxP3-Cre^–^ Bim^f/f^ (data not shown). Importantly, by 6 months of age the frequency of T_reg_ in FoxP3-Cre^+^Bim^f/f^ mice increased even further, reaching levels observed in Bim^−/−^ mice (Figure 2C). These data show that T_reg_ intrinsic loss of Bim is sufficient to promote T_reg_ accumulation with age.

### Bim is the major pro-apoptotic protein responsible for T_reg_ homeostasis

Although our data incriminate Bim as the major regulator of T_reg_ accumulation with age, we addressed the contribution of other pro-apoptotic proteins in T_reg_ survival, by using mice with T cell specific deletion of Bax and Bak, Lck-Cre^+^Bax^f/f^Bak^−/−^mice. Bax and Bak are the downstream mediators of pro-apoptotic proteins, and the deletion of both genes eliminates apoptosis through the intrinsic pathway (Youle and Strasser, 2008). If other pro-apoptotic proteins were critical in limiting T_reg_ survival, then T_reg_ accrual in Lck-Cre^+^ mice would be significantly greater than that observed in Bim^−/−^ mice. One caveat of comparing T_reg_ accrual between Lck-Cre^+^Bax^f/f^Bak^−/−^mice and Bim^−/−^mice is that non-T_reg_ loss of Bim could affect T_reg_ homeostasis; however, our data in FoxP3-Cre^+^Bim^f/f^ show that T_reg_-specific loss of Bim is sufficient to drive accrual to levels similar to those observed in Bim^−/−^ mice. T cell loss of Bax and Bak resulted in increased T_reg_ accumulation at 15 months of age compared to Lck-Cre^−^ mice (20% increase compared to 10% increase, respectively; Figure 2D). Importantly, the frequency of T_reg_ was similar in 15-month-old Lck-Cre^+^Bax^f/f^Bak^−/−^mice and age-matched Bim^−/−^mice, demonstrating that Bim is the major pro-apoptotic protein controlling T_reg_ survival.

### Both survival-dependent and – independent mechanism(s) contribute to the emergence of Bim^lo^ T_reg_

**Figure 3:**
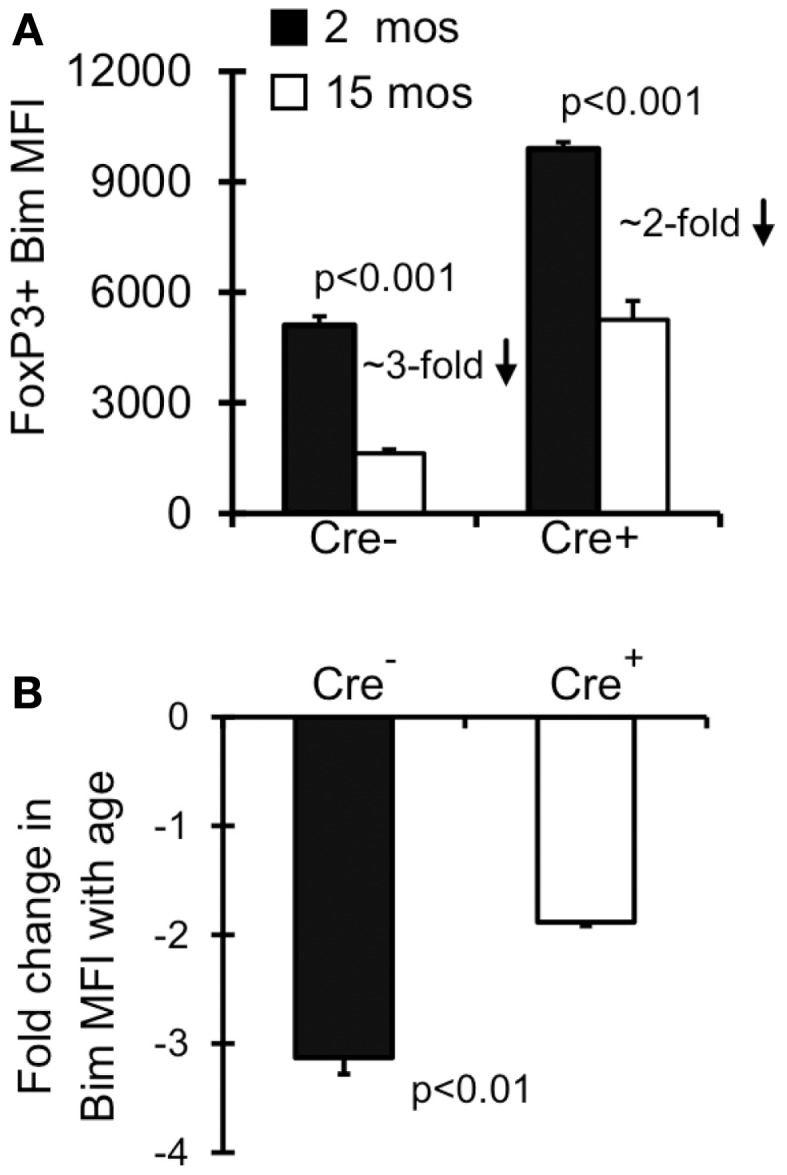
**Both environment and survival contribute to the emergence of Bim^lo^ T_reg_**. Splenocytes from Lck-Cre^−^Bax^f/f^Bak^−/−^(*n* = 3–5/group) and Lck-Cre^+^Bax^f/f^Bak^−/−^(*n* = 3–5/group) mice were stained for CD4, FoxP3, and Bim and analyzed by flow. **(A)** Results show the Bim MFI in CD4^+^FoxP3^+^ cells (±SE). The *p* values represent the difference between 2 month-old and 15 month-old mice (Student’s *t* test). **(B)** Results show the fold change in CD4^+^FoxP3^+^ Bim expression in 15 month Lck-Cre^−^Bax^f/f^Bak^−/−^and Lck-Cre^+^Baxf^/f^Bak^−/−^ mice compared to their 2 month counterparts. The *p* values represent the difference between Lck-Cre^−^ and Lck-Cre^+^ (Student’s *t* test).

Old Bim^lo^ T_reg_ have increased survival *ex vivo* (Chougnet et al., 2011); however, it remains unclear if T_reg_ with lower Bim expression emerge *in vivo* because they are afforded a selective survival advantage. To test this, we examined Bim levels in Lck-Cre^+^Bax^f/f^Bak^−/−^T_reg_ as their levels of Bim will be largely irrelevant to their survival. Notably, the levels of Bim were significantly increased in Lck-Cre^+^ mice compared to Lck-Cre^−^ mice, indicating T_reg_ are able to tolerate higher levels of Bim in the absence of Bax and Bak (Figure 3A). As expected, Bim levels in T_reg_ from 15-month-old Lck-Cre^−^ mice was significantly decreased compared to their 2-month-old counterparts (Figures 3A,B). Bim was also decreased in 15-month-old Lck-Cre^+^ mice (Figures 3A,B), but the fold-decrease in Bim expression in Lck-Cre^−^ mice was greater than in Lck-Cre^+^ mice (threefold vs. twofold, respectively; Figure 3B). Together, these data suggest that Bim levels are controlled in T_reg_ by both survival-dependent and independent mechanisms.

### An aged environment promotes the emergence of CD25^lo^ T_reg_

**Figure 4:**
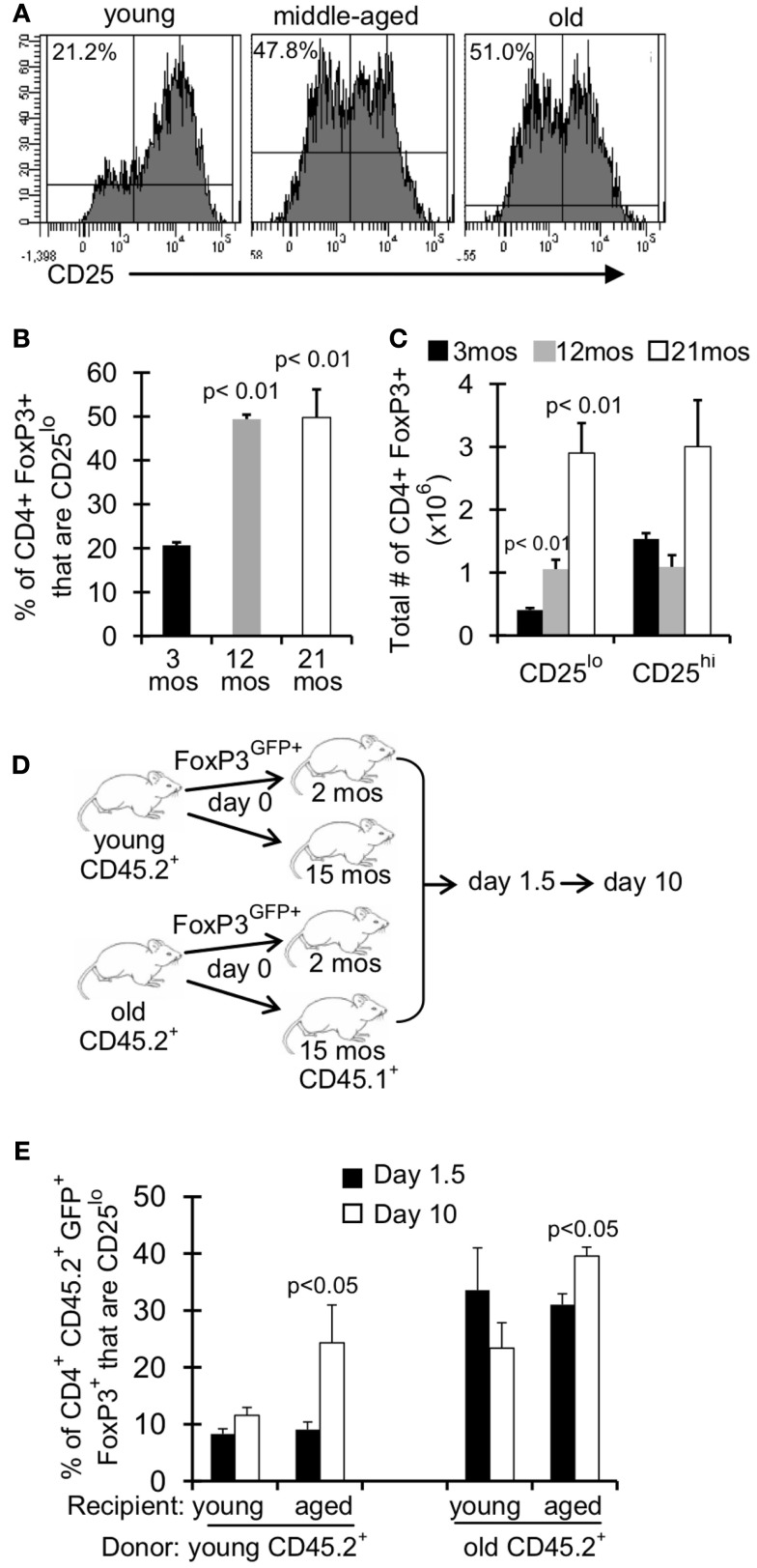
**An aged environment promotes emergence of CD25^lo^ T_reg_**. **(A–C)** Splenocytes from young (3 months; *n* = 4), middle-aged (12 months; *n* = 4), and old (21 months; *n* = 5) mice were stained for CD4, FoxP3, and CD25 and analyzed by flow. **(A)** Histograms show the expression of CD25 on CD4^+^FoxP3^+^ cells. Percentages are the frequencies of CD4^+^FoxP3^+^ that are CD25^lo^. **(B)** Results show the frequency of CD4^+^FoxP3^+^ cells that are CD25^lo^ (±SE). The *p* values represent the difference compared to 3-month-old mice (Student’s *t* test). **(C)** Results show the total number of CD4^+^FoxP3^+^ cells that are CD25^lo^ or CD25^hi^. The *p* values represent the difference compared to 3-month-old mice (Student’s *t* test). Results are representative of at least three independent experiments. **(D,E)** CD4^+^FoxP3^GFP+^ T cells were sorted from splenocytes of young (3–4 months) and old (>18 months) CD45.2 FoxP3^GFP^ reporter mice. About 5 × 10^5^ CD4^+^FoxP3^GFP+^ cells were injected i.v. into young (2 months) and aged (15 months) congenic CD45.1 mice. Mice were sacrificed at day 1.5 (*n* = 3–5/group) and day 10 (*n* = 3–5/group) post-injection. Cells from the spleen were stained for CD4, FoxP3, CD25, and CD45.2 and analyzed by flow. **(E)** Results show the frequency of CD4^+^CD45.2^+^FoxP3^+^GFP^+^ cells that are CD25lo (±SE). The *p* values represent the difference between day 1.5 and 10 (Student’s *t* test).

**Figure A1:**
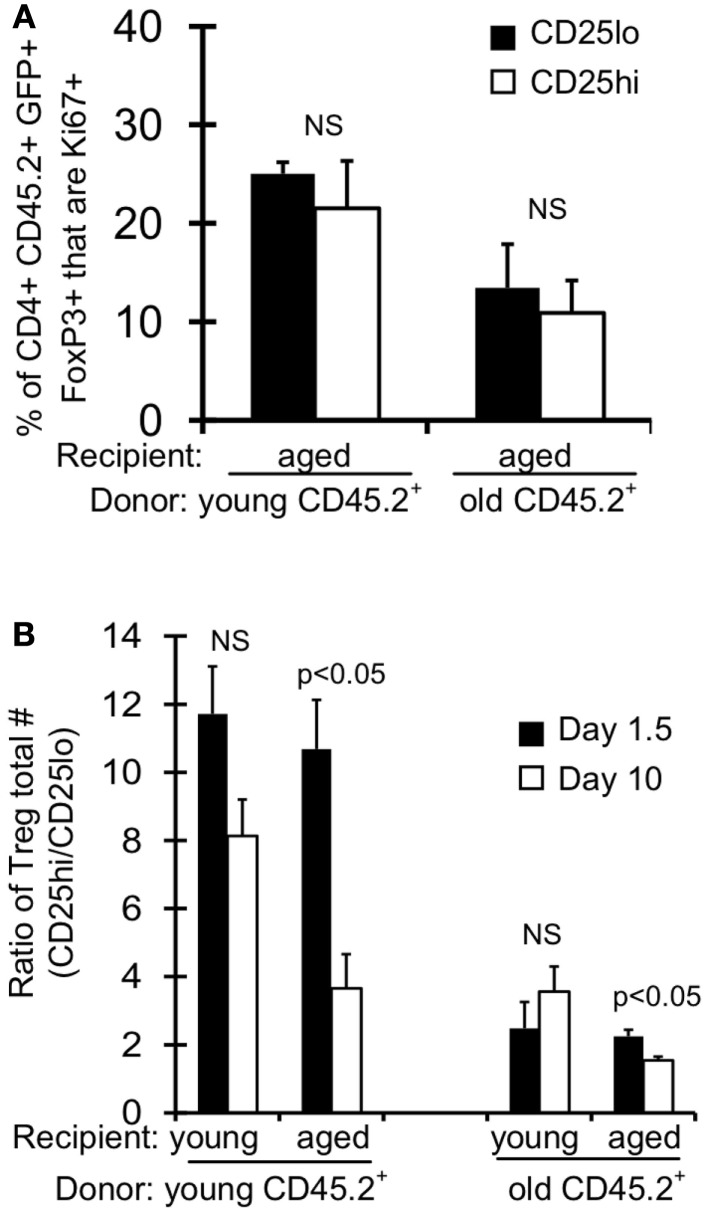
**Absolute numbers and proliferation of Treg post-adoptive transfer**. CD^+^FoxP3^GFP+^ T cells were sorted from splenocytes of young (3–4 months) and old (>18 months) CD45.2 FoxP3^GFP^ reporter mice. About 5 × 10^5^ CD4^+^FoxP3^GFP+^ cells were injected i.v. into young (2 months) and aged (15 months) congenic CD45.1 mice. Mice were sacrificed at day 1.5 (*n* = 3–5/group) and day 10 (*n* = 3–5/group) post-injection. Cells from the spleen were stained for CD4, CD25, CD45.2, and intracellularly for FoxP3 and Ki67 and analyzed by flow. **(A)** Results show the frequency of CD4^+^CD45.2^+^FoxP3^+^GFP^+^CD25^lo^ and CD25^hi^ cells that are Ki67^+^ on day 10 post-transfer into aged recipient mice (±SE). **(B)** Results show the numbers ratio of CD25^hi^ to CD25^lo^ CD4^+^CD45.2^+^FoxP3^+^GFP^+^ cells (±SE).

T_reg_ are described in the literature as CD4^+^FoxP3^+^ CD25^hi^, however there is a substantial population of CD4^+^FoxP3^+^ CD25^lo^ cells in young mice ( ∼10–20% of FoxP3^+^ cells; Figures 4A,B). Interestingly, this population of CD25^lo^ T_reg_ expands with age, comprising 50% of the T_reg_ population by middle-aged (Figures 4A,B) (Nishioka et al., 2006; Lages et al., 2008). Importantly, while both CD25^lo^ and CD25^hi^ T_reg_ expand with age, the relative increase in CD25^lo^ T_reg_ is much greater (Figure 4C). To assess whether the aged environment drives CD25^lo^ T_reg_ accumulation, we adoptively transferred young (3–4 months) and old (19–23 months) T_reg_ into young (2 months) and aged (15 months) recipient mice (Figure 4D). At 1.5 and 10 days later, recipient mice were sacrificed and the frequency of CD25^lo^ T_reg_ from donors was assessed (Figure 4E). Interestingly, when young cells were placed into aged recipients, we observed an emergence of CD25^lo^ T_reg_. Similarly, CD25^lo^ T_reg_ were enriched slightly if old T_reg_ were transferred into aged mice. Increases in CD25^lo^ T_reg_ are likely not due to increased proliferation as there was an equivalent percentage of CD25^lo^ and CD25^hi^ T_reg_ that were Ki67^+^ after transfer into aged mice (Figure A1A in Appendix). Further, the total numbers of CD25^lo^ T_reg_ were affected as the ratio of CD25^hi^ to CD25^lo^ T_reg_ decreased by day 10 post-transfer (Figure A1B in Appendix), suggesting that CD25^hi^ T_reg_ may lose expression of CD25. On the other hand, when young or old T_reg_ were placed into young donors, we did not see an enrichment of CD25^lo^ T_reg_. Together, these data strongly suggest that environmental changes with age support the emergence of CD25^lo^ T_reg_.

### Declining IL-2 levels with age fosters the emergence of CD25^lo^Bim^lo^ T_reg_

**Figure 5:**
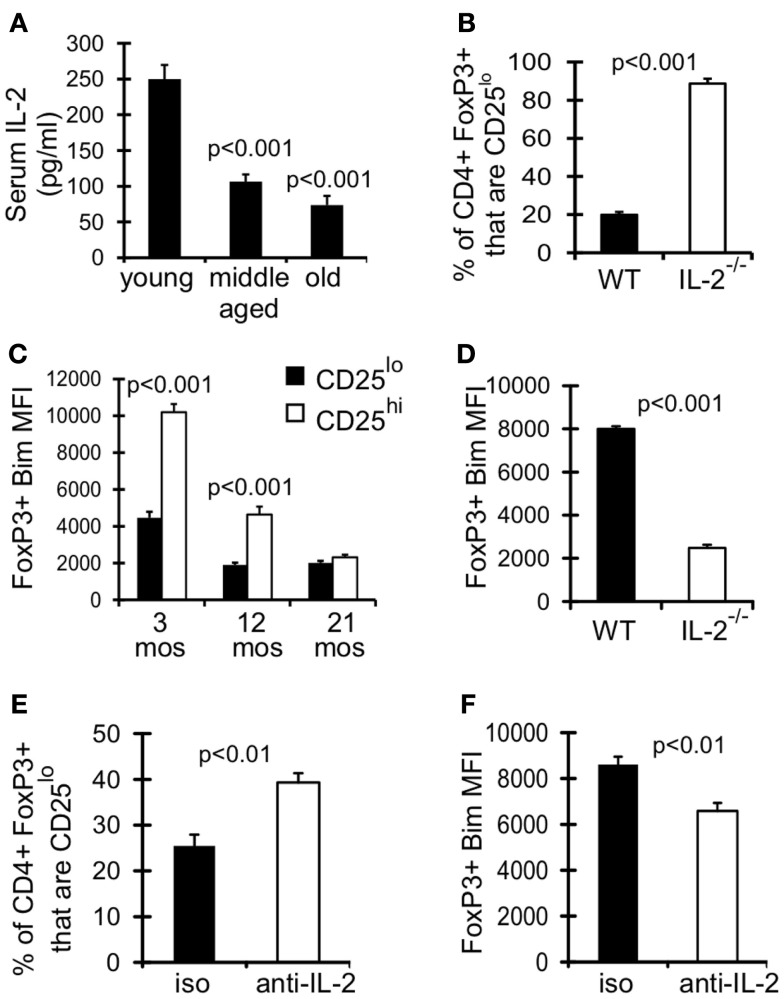
**Declining IL-2 levels drives emergence of CD25^lo^Bim^lo^ T_reg_**. **(A)** Serum IL-2 was determined by IVCCA. Results show the average serum IL-2 levels from young (3–4 months; *n* = 10), middle-aged (11–12 months; *n* = 11), and old (>15 months; *n* = 9) mice (±SE). Results are representative of 3 independent experiments. The *p* values represent the difference compared to young (Student’s *t* test). **(B,D)** Splenocytes from 5-week-old C57BL/6 wild-type (WT; *n* = 4) and IL-2^−/−^ (*n* = 3) mice were stained for CD4, FoxP3, CD25, and Bim and analyzed by flow. **(B)** Results show the frequency of CD4^+^FoxP3^+^ cells that are CD25lo (±SE) and are representative of two independent experiments. **(C)** Splenocytes from 3 month (*n* = 4), 12 month (*n* = 4), and 21-month-old (*n* = 5) mice were stained for CD4, FoxP3, CD25, and Bim and analyzed by flow. Results show the Bim MFI of CD4^+^FoxP3^+^CD25^lo^ cells and CD4^+^FoxP3^+^CD25^hi^ cells (±SE). The *p* values represent the difference between CD25^lo^ and CD25^hi^ T_reg_ (Student’s *t* test). **(D)** Results show the Bim MFI of CD4^+^FoxP3^+^ T_reg_ from WT and IL-2^−/−^ mice (±SE) and are representative of two independent experiments. **(E,F)** Three-month-old mice were injected with IL-2 neutralizing antibodies or IgG isotype control (*n* = 4/group). Spleens were harvested at day 7 and stained for CD4, FoxP3, CD25, and Bim. **(E)** Results show the frequency of CD4^+^FoxP3^+^ cells that are CD25^lo^ (±SE). **(F)** Results show the Bim MFI of CD4^+^FoxP3^+^ T_reg_ (±SE).

IL-2 signaling is known to promote expression of its own receptor CD25 (IL-2Rα) (Liao et al., 2013), and could be critical for maintaining CD25^hi^ T_reg_. As assessed in the serum, levels of IL-2 were significantly decreased in middle-aged and old mice compared to young mice (Figure 5A). Furthermore, T_reg_ in IL-2^−/−^ mice were comprised almost entirely of CD25^lo^ cells (Figure 5B). Interestingly, CD25^lo^ T_reg_ were Bim^lo^ at any age (Figure 5C), and this lower expression of Bim may promote their increased accrual with age. To assess whether IL-2 also controls Bim expression we measured Bim levels in T_reg_ from IL-2^−/−^ mice. Strikingly, Bim levels were significantly decreased in T_reg_ from IL-2^−/−^ mice (Figure 5D). This loss of Bim expression occurred in the periphery, as Bim levels in thymic T_reg_ were similar between IL-2^−/−^ and wild-type mice (data not shown). To independently test the role of IL-2 in maintaining CD25^hi^Bim^hi^ T_reg_, we neutralized IL-2 in young mice (3 months), which resulted in decreased expression of Bim and an enrichment of CD25^lo^ T_reg_ (Figures 5E,F). Also, the total number of T_reg_ were not decreased after IL-2 neutralization, suggesting that CD25^hi^ T_reg_ lose expression of CD25 (data not shown). Taken together, these data show that IL-2 is critical for maintaining T_reg_ with a CD25^hi^Bim^hi^ phenotype, both in young and aged mice. These data also suggest that reduced IL-2 with age promotes the emergence of CD25^lo^Bim^lo^ cells.

### IL-15 contributes to T_reg_ homeostasis in middle-aged mice

**Figure 6:**
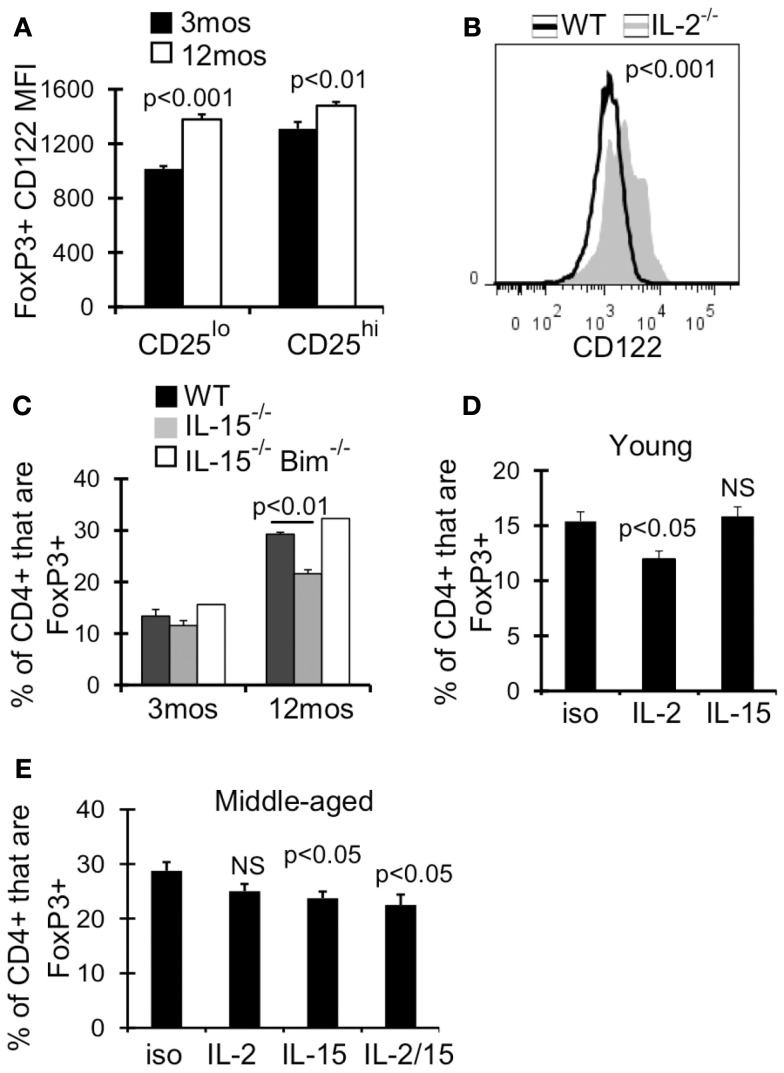
**IL-15 antagonizes Bim to promote T_reg_ homeostasis in middle-aged mice**. Splenocytes from 3 month (*n* = 4) and 12-month-old (*n* = 4) mice **(A)** or 5-week-old WT (*n* = 4) and IL-2^−/−^ (*n* = 3) mice **(B)** were stained for CD4, FoxP3, and CD122, and analyzed by flow. **(A)** Results show the MFI of CD122 on CD4^+^FoxP3^+^ cells (±SE). **(B)** Histograms show CD122 expression on CD4^+^FoxP3^+^ cells. The *p* value represents the difference between the average CD122 MFI from WT and IL-2^−/−^ mice (Student’s *t* test). **(C)** Splenocytes from WT (*n* = 3–4/group), IL-15^−/−^ (*n* = 3/group), and IL-15^−/−^ Bim^−/−^ (*n* = 2/group) were stained for CD4 and FoxP3 and analyzed by flow. Results show the frequency of CD4^+^ cells that are FoxP3^+^ (±SE) and are representative of two independent experiments. Young (3 months; *n* = 4/group) **(D)** and middle-aged (12 months; *n* = 5/group) **(E)** mice were injected with IL-2 and/or IL-15 neutralizing antibody or with IgG isotype control. Spleens were harvested at day 7 and stained for CD4, FoxP3, CD25, and Bim. **(D,E)** Results show the frequency of CD4^+^ cells that are FoxP3^+^ (±SE). The *p* values represent the difference between antibody treated and isotype control (Student’s *t* test).

We next examined the expression of receptors for other common γ cytokines which can function redundantly with IL-2 to support T_reg_ homeostasis and survival in young mice (Fontenot et al., 2005a; Burchill et al., 2007; Bayer et al., 2008; Vang et al., 2008). Although the expression of IL-7Rα (CD127) increases with age, we previously showed that blocking CD127 signaling alone did not affect T_reg_ survival in 12-month-old mice (Chougnet et al., 2011). IL-2Rβ (CD122) is a part of both the IL-2 and IL-15 receptor, and its expression was significantly upregulated on T_reg_ by 12 months of age (Figure 6A). Additionally, CD122 expression was increased twofold on T_reg_ in IL-2^−/−^ mice (AVG MFI: WT = 1265; IL-2^−/−^ = 2378; *p* < 0.001) (Figure 6B). We therefore assessed the role of IL-15 in T_reg_ accumulation in middle-aged mice, as increased T_reg_ CD122 expression and increased CD25^lo^ T_reg_ by 12 months of age may reflect a change in T_reg_ cytokine dependency. While there was no difference in T_reg_ frequency in young IL-15^−/−^ and wild-type mice, the frequency of T_reg_ was significantly reduced in IL-15^−/−^ mice by 12 months of age (Figure 6C). Importantly, the additional loss of Bim (IL-15^−/−^Bim^−/−^), rescued the loss of T_reg_ in middle-aged IL-15^−/−^ mice (Figure 6C), suggesting that IL-15 supports T_reg_ homeostasis in aged mice by combating Bim-mediated cell death.

To test whether IL-15 promotes T_reg_ survival when IL-2 levels decline, we neutralized IL-2 and/or IL-15 in young and middle-aged mice. IL-2 neutralization in young mice (3 months) but not middle-aged mice (12 months) resulted in a significant loss of T_reg_ (Figures 6D,E), supporting that IL-2 is less critical for T_reg_ homeostasis in an aged environment. Conversely, IL-15 neutralization in middle-aged mice resulted in a significant loss of T_reg_, which was not observed in young mice (Figures 6D,E). Importantly, neutralization of IL-2 and IL-15 did not result in further loss of T_reg_ compared to IL-15 neutralization alone (Figure 6E). Together, our data suggests that in an aging environment, IL-15 supports T_reg_ survival in the face of declining IL-2.

## Discussion

Age-related immunosuppression is multi-factorial, encompassing cell-intrinsic defects within T and B cells, as well as population based defects such as a loss of naïve T cells and increased levels of regulatory T cells (Valmori et al., 2005; Nishioka et al., 2006; Sharma et al., 2006; Lages et al., 2008; Agius et al., 2009). Increased levels of T_reg_ are observed in both mice and humans and functionally suppress T cell responses to both parasites (*L. major*) and tumors (Sharma et al., 2006; Lages et al., 2008). We also recently showed that Bim is a key controller of T_reg_ homeostasis as T_reg_ accrual is accelerated in Bim-deficient (Bim^−/−^) mice (Chougnet et al., 2011); although it was unclear if this was due to T cell specific effects of Bim in peripheral T_reg_. Here, we focused on further understanding the mechanisms controlling T_reg_ accrual in aging.

First, the germline deletion of Bim results in loss of Bim from all tissues and it was possible that non-T cell expression of Bim controls T_reg_ homeostasis. In particular, the increased lifespan of Bim-deficient dendritic cells (DC) (Chen et al., 2007) could contribute to T_reg_ accrual as DC can promote T_reg_ homeostasis and inducible T_reg_ development (Yamazaki et al., 2003; Belkaid and Oldenhove, 2008). Second, Bim^−/−^ thymocytes are resistant to negative selection (Bouillet et al., 2002) and such altered thymic development in Bim^−/−^ mice could skew the levels of T_reg_ in Bim^−/−^ mice. By using FoxP3-Cre, which would likely not turn on until after cells have committed to the T_reg_ lineage and largely at a point beyond negative selection (Fontenot et al., 2005b), we avoided the effects of Bim in other tissues and the effects of Bim on thymic development. Thus, our data clearly show that T_reg_-specific loss of Bim is sufficient to drive T_reg_ accrual with age and strongly suggest that the normal decline of Bim expression seen in wild-type mice promotes T_reg_ accrual in aging mice.

While our data support Bim as a major controller of T_reg_ homeostasis, we could not exclude potentially redundant roles for other pro-apoptotic Bcl-2 family members. For instance, the additional loss of Puma in Bim-deficient mice enhances T cell survival, suggesting a redundant role for Puma with Bim in certain T cell contexts (Erlacher et al., 2006; Gray et al., 2012). Moreover, we have found that, like Bim, Puma levels are normally decreased as mice age (data not shown). However, mice singly deficient in either Bmf, Puma, Noxa, or Bad have no difference in T_reg_ frequencies in young mice (Tischner et al., 2012), while young Bim-deficient mice have subtle, but significant increases in T_reg_ (Zhan et al., 2011; Tischner et al., 2012). Further, our finding that T_reg_ accrual was similar in aged Bim^−/−^ mice and Lck-Cre^+^Bax^f/f^Bak^−/−^mice strongly suggest that Bim is the main negative regulator of T_reg_ homeostasis with age.

IL-2, on the other hand, is a major positive regulator of T_reg_ homeostasis as IL-2-deficient mice have significantly reduced T_reg_ (Fontenot et al., 2005a; Burchill et al., 2007; Barron et al., 2010). Although it has been accepted for many years that IL-2 levels decrease with age, this assumption was largely based upon a reduced production of IL-2 after *in vitro* stimulation of aged T cells (Thoman and Weigle, 1981). To the best of our knowledge, our study is the first demonstration that *in vivo* IL-2 levels are decreased in aged mice. We acknowledge that serum IL-2 levels may not fully reflect the *in vivo* bioavailability of IL-2, as IL-2 can bind to heparin sulfate on extracellular matrices (Wrenshall and Platt, 1999), but we did not observe substantially increased staining for IL-2 on spleen tissue from old mice (data not shown). We previously proposed that continued IL-2 signaling drives the expansion of Bim^lo^ T_reg_, as exogenous IL-2 preferentially induced proliferation and expansion of Bim^lo^ T_reg_ (Chougnet et al., 2011). We note that these experiments were performed in young mice that were given supraphysiologic levels of IL-2. Given that we now know that IL-2 levels decline by middle-age, this model seems less likely. Further, our new data clearly show that neutralization of IL-2 and/or loss of IL-2 both lead to the accumulation of CD25^lo^ T_reg_ and decreased the levels of Bim within these cells. It does seem paradoxical that reduced levels of IL-2 in aged mice would be associated with T_reg_ accrual. However, our and others data suggest that IL-2 and Bim antagonize one another to regulate T_reg_ homeostasis. For example, the absence of Bim can restore T_reg_ in IL-2-deficient mice (Barron et al., 2010) and we found that limiting or restricting IL-2 results in the accrual of Bim^lo^ T_reg_. Thus, while it has been shown in cell lines that acute withdrawal of IL-2 leads to increased Bim expression (Stahl et al., 2002), chronic limiting IL-2 may select for Bim^lo^ T_reg_. Further, we show that the lack of IL-15 reduces the age-related accumulation of T_reg_ and that the additional loss of Bim restores T_reg_ accrual. We did not observe an effect of IL-15 on Bim expression directly, but we cannot exclude that IL-15 acts to restrain the remaining levels of Bim through another mechanism (i.e., Bim phosphorylation and turnover, induction of another anti-apoptotic molecule). As IL-2 and IL-15 both signal through CD122 (IL-2/15Rβ) and CD132 (common γ chain), it is possible that the decline of IL-2 favors the emergence of CD25^lo^Bim^lo^ T_reg_ because these cells are selected to survive and are maintained by IL-2-independent mechanisms, such as IL-15 (Fontenot et al., 2005a; Burchill et al., 2007).

While IL-2 and IL-15 signal through the same receptors, they can have divergent effects on T cells. For example, both IL-2 and IL-15 can activate the STAT5 and PI-3K/AKT pathways (Waldmann, 2006). In CD8^+^ T cells, the magnitude of STAT5 activation by IL-2 and IL-15 is similar, although IL-2 drives more prolonged STAT5 activation (Castro et al., 2011). Further, while both IL-2 and IL-15 can activate PI-3K/AKT/mTOR, IL-2 drives both an increased magnitude and persistence of mTOR activation compared to IL-15 (Cornish et al., 2006; Castro et al., 2011). Recent work has shown that unrestrained activation of mTORC1 in T cells lead to significantly increased Bim expression (Yang et al., 2011). Thus, it is possible that the differential activation of mTORC1 by IL-2 and IL-15 may underlie Bim regulation in T_reg_. However, it remains to be determined whether or not the degree to which the mechanisms are operative in T_reg_ cells.

Bim is also regulated at the transcriptional level by the FOXO family of transcription factors. FOXO transcription factors promote Bim expression in T cells in response to cytokine withdrawal (Stahl et al., 2002; Salih and Brunet, 2008). FOXO(s) are inhibited by PI-3K/AKT activation, which leads to FOXO phosphorylation and sequestration from the nucleus (Salih and Brunet, 2008). Consistent with this, we found that *in vivo* IL-2 administration to young mice resulted in accrual of Bim^lo^ T_reg_ (Chougnet et al., 2011). However, in aged mice we found decreased *in vivo* IL-2 levels and *decreased* Bim expression, which are inconsistent with a role for FOXO transcription factor control of Bim. Interestingly, we also find that FOXO1 and FOXO3a levels themselves are decreased in aged T_reg_ (data not shown). As mentioned earlier, levels of Puma, another FOXO target are also decreased in aged T_reg_, raising the possibility that decreased levels of FOXO molecules may contribute to decreased expression of Bim.

Certainly, a survival advantage contributes to the accrual of T_reg_ with low Bim expression as T_reg_ with higher levels of Bim accumulate in mice whose T_reg_ cannot undergo apoptosis (i.e., T cell-specific Bax/Bak-deficient mice). One factor that may promote selection of Bim^lo^ T_reg_ is the decreased expression of Bcl-2 and Mcl-1 observed in aged T_reg_ (Chougnet et al., 2011). Indeed, Bcl-2 is the major anti-apoptotic reported to combat Bim-mediated death in T cells (Wojciechowski et al., 2007), and Bim and Bcl-2 have been shown to affect the expression of each other (Jorgensen et al., 2007). Further, we recently showed in CD8^+^ T cells that inhibition or loss of Bcl-2 selected for effector CD8^+^ T cells expressing low levels of Bim (Kurtulus et al., 2011). Thus, decreased expression of Bcl-2 with age may prompt the selection of Bim^lo^ T_reg_. However, it is also clear that, relative to their young counterparts, Bim levels decline even in T cell-specific Bax/Bak-deficient mice, suggesting that levels of Bim may be controlled by non-survival related mechanisms as well. An alternative, and not mutually exclusive, explanation is that decreased Bim mRNA levels in aged T_reg_ may be due to epigenetic modification of the Bim promoter. The Bim promoter is CpG rich, and there is evidence that Bim expression can be repressed through hypermethylation (Paschos et al., 2009; San Jose-Eneriz et al., 2009; De Bruyne et al., 2010; Richter-Larrea et al., 2010). Furthermore, increased CpG methylation is a trait observed in aged cells (Golbus et al., 1990; Issa, 2003). Thus, as epigenetic modifications can be inherited, Bim promoter methylation may aid in the prolonged survival of aged T_reg_. We are currently determining whether epigenetic and/or transcriptional repression affects Bim expression in aged T_reg_.

While the exact mechanisms leading to decreased T_reg_ Bim expression with age are still unclear, progressive loss of Bim expression occurs in both CD25^lo^ and CD25^hi^ T_reg_ and this loss is likely sufficient to drive accrual of both populations with age. Furthermore, it is likely the lower expression of Bim in CD25^lo^ T_reg_ that affords these cells a survival advantage over CD25^hi^ T_reg_, especially when IL-2 becomes limiting, and promotes increased CD25^lo^ T_reg_ accrual. Indeed, not until IL-2 levels have declined do we begin to see the emergence of CD25^lo^ T_reg_ in middle-aged mice. Because IL-2 promotes the expression of CD25 (Liao et al., 2013), there is likely still enough bioavailable IL-2 present in aged mice to maintain CD25 expression on the accumulated CD25^hi^ T_reg_ population. Thus, it is likely that both reduced IL-2 signaling as well as other mechanism(s) (i.e., Bim promoter epigenetics) contribute to the control of Bim expression with age.

In this study, we show that T_reg_ loss of Bim drives accrual with age, while IL-15 is critical for aged T_reg_ survival in the face of declining IL-2. We have shown here that T_reg_ accrual is driven by several non-mutually exclusive mechanisms, including: (i) decreased Bim transcription, (ii) decreased Bim protein half-life, (iii) selection of Bim^lo^ T_reg_ due to a survival advantage, and (iv) a switch in cytokine dependency (from IL-2 to IL-15) with age. Future studies will focus on the mechanisms driving decreased Bim gene expression and protein half-life (i.e., promoter epigenetics, AKT/FOXO pathway), which may expose potential therapeutic targets for manipulating Bim expression and T_reg_ accrual. The ability to moderately manipulate T_reg_ homeostasis and accrual, without inducing inflammation and autoimmune responses, may provide a potential therapy for enhancing immune competence in the elderly.

## Conflict of Interest Statement

The authors declare that the research was conducted in the absence of any commercial or financial relationships that could be construed as a potential conflict of interest.
